# Ethmoid glomangioma and oncogenic osteomalacia: a case report

**DOI:** 10.1186/s13256-021-02916-0

**Published:** 2021-07-17

**Authors:** Camila R. Muniz, Gabriela A. M. Bezerra, Viviane C. da Silva, Priscilla M. F. Aguiar, Gunter Gerson, Catarina B. D’Alva, André A. A. Nunes

**Affiliations:** 1 Department of Otorhinolaryngology, Walter Cantídio University Hospital of Ceará Federal University, Capitão Francisco Pedro Street, 1290, Ilha 6, Rodolfo Teófilo, Fortaleza, CE CEP: 60430-370 Brazil; 2 Department of Pathology, Walter Cantídio University Hospital of Ceará Federal University, Fortaleza, Brazil; 3 Department of Endocrinology, Walter Cantídio University Hospital of Ceará Federal University, Fortaleza, Brazil

**Keywords:** Oncogenic osteomalacia, Glomangioma, Paranasal sinus tumor

## Abstract

**Background:**

Glomangioma is a benign tumor of mesenchymal origin, derived from the glomus body. It is responsible for the thermal regulation of the dermis. The occurrence of oncogenic osteomalacia related to glomangioma is rare. Only two cases have been reported thus far.

**Case presentation:**

A 32-year-old female, Brazilian, presented diffuse pain, during pregnancy, that developed progressively, limiting her mobility. Imaging showed a femoral neck fracture, and rheumatological laboratory examination showed hypophosphatemia. Also, the patient reported episodes of epistaxis during childhood and recurrence along with progressively right nasal obstruction. Endoscopic resection of the tumor was performed, and immunohistochemistry was conclusive for glomangioma. This case report describes the third case in which endonasal endoscopic surgery resulted in a favorable outcome.

**Conclusion:**

This case of glomangioma-induced oncogenic osteomalacia suggests that surgeons and clinicians should consider sinonasal tumors as a differential diagnosis of osteomalacia, and endonasal endoscopic surgery should be a possible curative resection.

## Background

Glomangioma is a benign tumor of mesenchymal origin, derived from the glomus body, which is neural and muscular structure that helps to thermally regulate the dermis [1]. It originates from proliferating arteriovenous capillary anastomoses and represents hyperplasia or hamartomatous development of the glomus body [2].

It is common in extremities such as the feet, toes, and palmar surface as well as the subungual and subcutaneous regions. Only 0.6% of all nonepithelial tumors of the nasal cavity, nasopharynx, and paranasal sinus result from glomangioma [1–4].

About 500 cases of oncogenic osteomalacia (OO) have been described in the literature [5]. Twelve cases of mesenchymal tumors of the ethmoid sinus associated with OO have been reported [6]. OO related to glomangioma is rare and has been associated with glomangiopericytomas or other soft-tissue tumors and bone tumors. We reviewed the literature and found only two reported cases; this is the first case of OO related to glomangioma documented in Brazil [1, 7–9].

## Case report

**Fig. 1:**
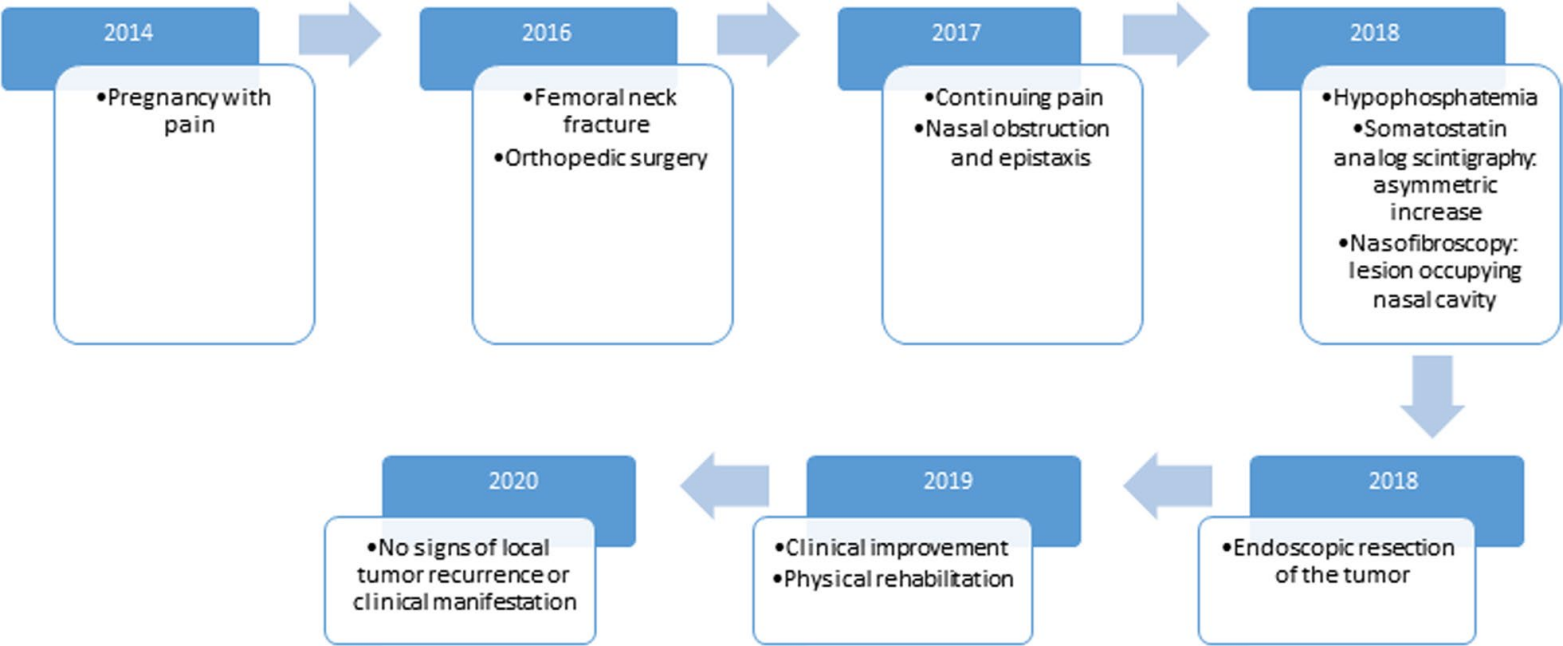
Timeline of clinical and treatment events: upper blue box describes year, and white box immediately below describes corresponding information

A 32-year-old female, Brazilian, presented with diffuse pain, predominantly in the upper and lower limbs and back. These symptoms had begun during pregnancy but did not compromise it, and fetal development was normal. The disease developed slowly and progressively, limiting her mobility 2 years after pregnancy. Magnetic resonance imaging showed a femoral neck fracture. Orthopedic surgery was performed. Due to the continuing pain, other specialists, including a neurologist and an endocrinologist, were consulted. She was diagnosed with fibromyalgia and osteoporosis. Rheumatological laboratory examination showed hypophosphatemia and normal level of 1,25(OH)_2_D (Fig. 1).

**Fig. 2:**
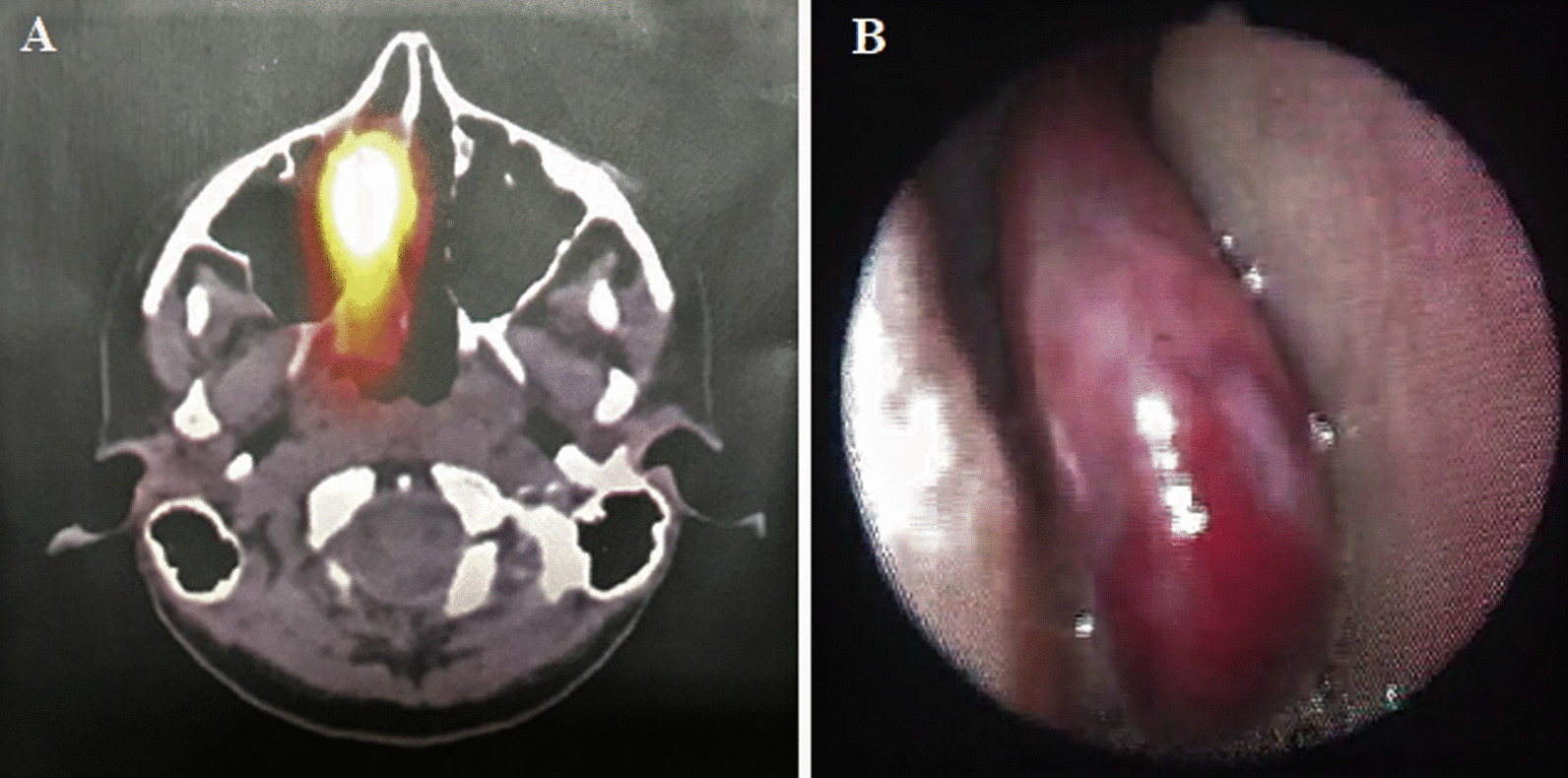
**A** Somatostatin analog scintigraphy: asymmetric increase of the radiopharmaceutical concentration in the right nasopharynx; **B** nasofibroscopy: reddish lesion occupying the right nasal cavity from the sphenoethmoidal recess

**Fig. 3:**
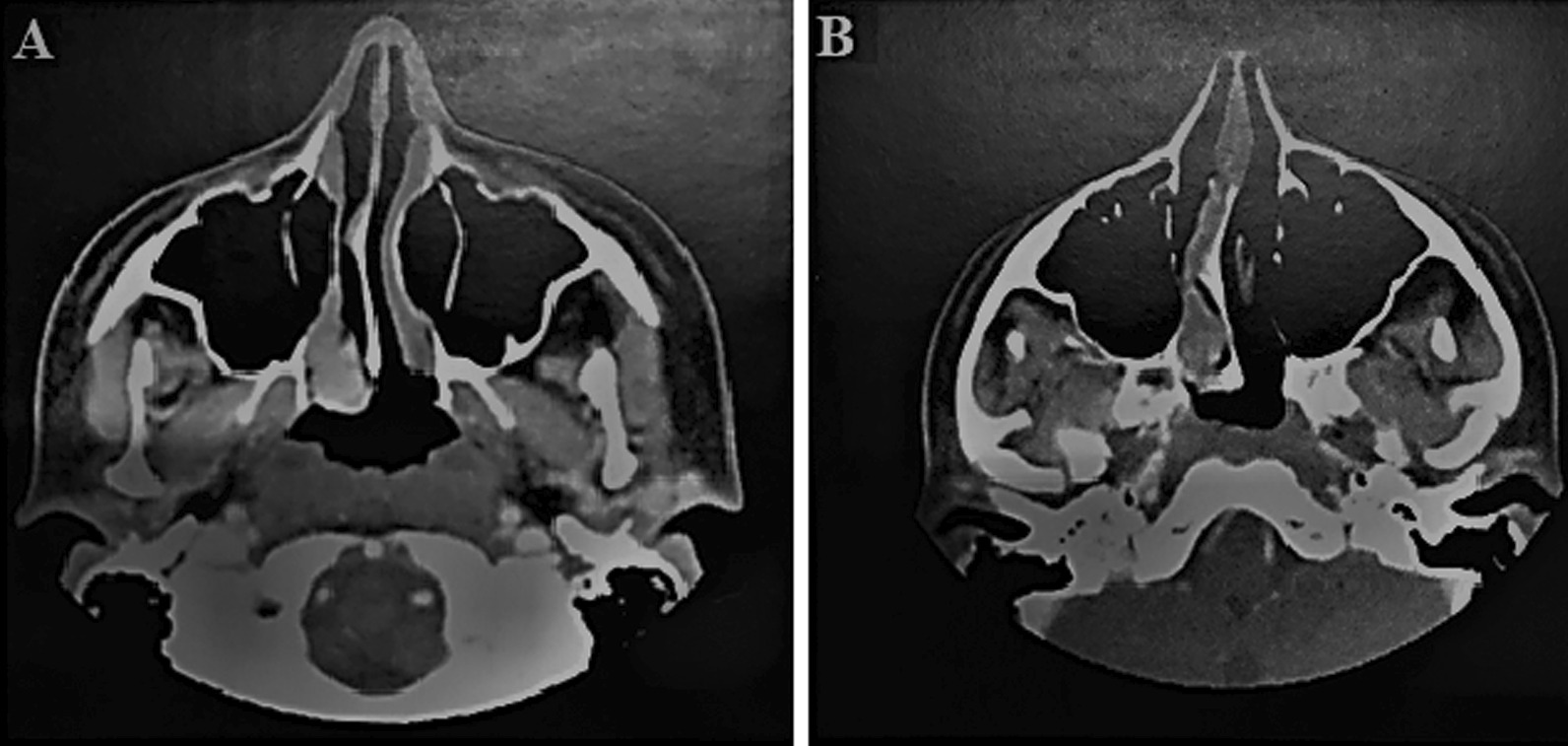
**A**, **B** Expansive contrast-enhancing polypoid lesion originating at the confluence between the nasal cavity roof, sphenoethmoidal recess, and right posterior ethmoid cell, measuring 1.5 × 2.0 × 5.1 cm

In the right nasopharynx, somatostatin analog scintigraphy revealed an asymmetric increase in radiopharmaceutical concentration (Fig. 2a). Furthermore, otolaryngological evaluation was requested. The patient reported episodes of occasional epistaxis during childhood and a recurrence along with a progressively right nasal obstruction. Nasofibroscopy showed a reddish lesion occupying the right nasal cavity from sphenoethmoidal recess (Fig. 2b). Computed tomography showed an expansive contrast-enhancing polypoid lesion originating at confluence between nasal cavity roof, sphenoethmoidal recess, and right posterior ethmoid cell, measuring 1.5 × 2.0 × 5.1 cm (Fig. 3a, b).

**Fig. 4:**
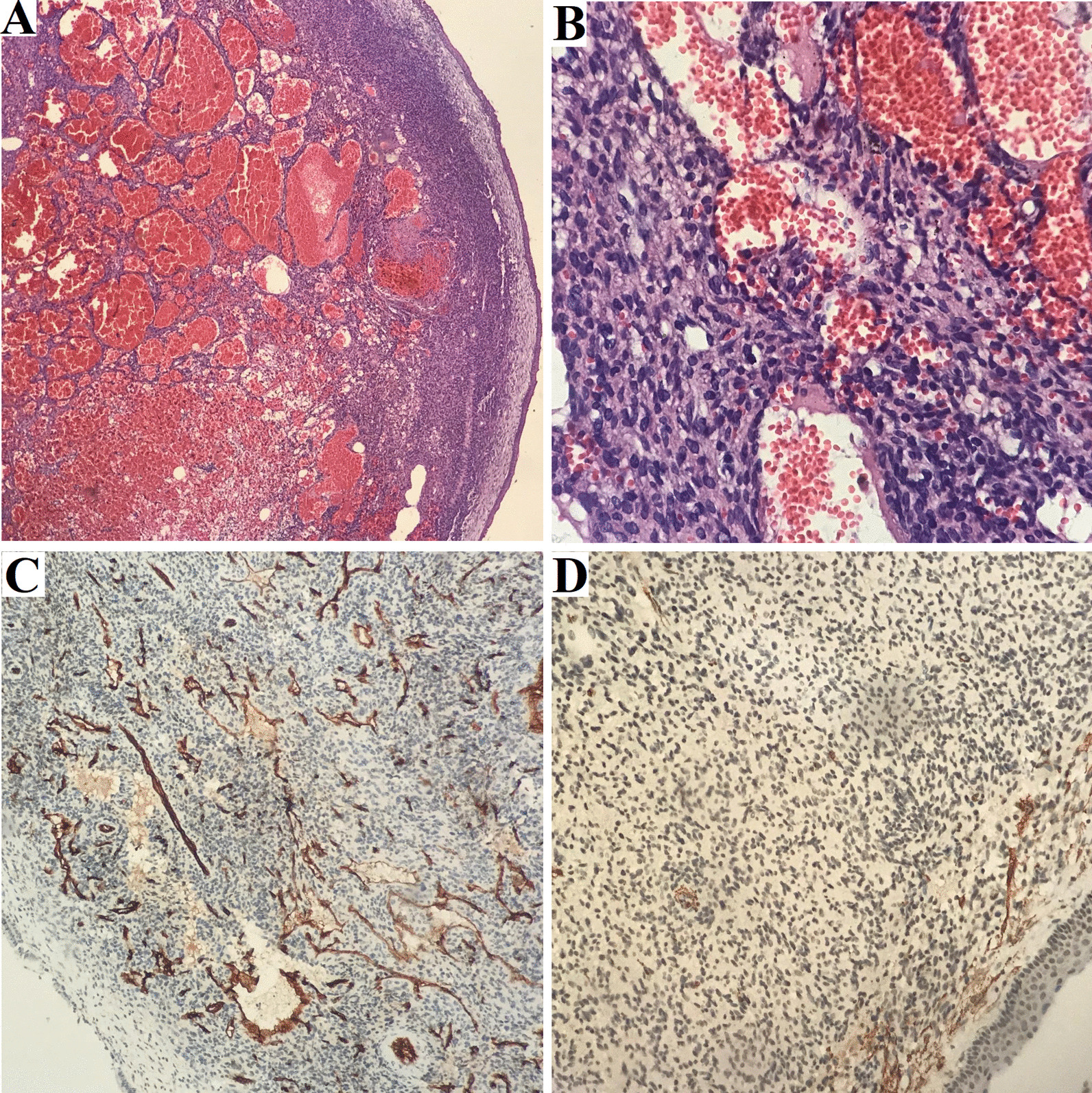
Microphotograph hematoxylin and eosin (HE): **A** respiratory mucosa with ectatic vessel proliferation, ×4; **B** fine-caliber ectatic vessels with associated elongated and spindle-shaped nuclei cells arranged in diffuse beams, ×40; **C** CD34, clone QBEnd/10—focal positive, ×10; **D** smooth muscle actin, clone 1A4—focal positive, ×20

Endoscopic resection of tumor was performed with right maxillary, sphenoid, frontal, and ethmoid sinusotomy. Surgery occurred without complications. After surgery, the patient was administered antibiotics and nasal irrigation with saline solution, and did not exhibit nasal symptoms. Anatomopathological examination revealed fine-caliber ectatic vessels associated with elongated and spindle-shaped cells, arranged in diffuse beams and permeated (Fig. 4). The immunohistochemistry report was conclusive for glomangioma, with positive results in CD34, and smooth muscle actin (SMA) (Fig. 4). Clinical improvement was observed during physical rehabilitation. There was an increase in serum phosphorus levels. Patient’s follow-up included clinical evaluation, nasal endoscopy, and laboratory tests. Two years after surgery, no signs of local tumor recurrence or hypophosphatemia clinical manifestation was observed. Clinical and treatment events are represented in a timeline (Fig. 1).

## Discussion

Glomangioma is a mesenchymal tumor that is derived from the glomus body and is present throughout the body. It represents hyperplasia or hamartomatous development of the glomus body. It is rare in the head and neck region. The first case of nasal glomangioma was reported in 1965. Since then, only 31 cases have been reported in the literature [1–4].

Glomangioma presents as a reddish or purplish polypoid mass located in nasal cavity, paranasal sinuses, or nasal septum. It is less frequently present in ethmoid sinus and usually affects the elderly, although there have been reports in younger people, as in this case [3, 10]. Females are affected more than men (1.9:1) [4].

Symptoms include nasal obstruction, epistaxis, and pain [3]. This patient reported having two of the three most common symptoms. Cases of asymptomatic presentation occur less frequently [4].

About 500 cases of OO have been described in the literature [5]. Twelve cases of ethmoidal sinus mesenchymal tumors associated with OO have been reported in the literature [6]. It is rarely related to glomangioma. Glomangiopericytomas and others mesenchymal tumors have been associated with it [1, 7–9]. Of the two cases reported thus far, this is the first documented in Brazil [1].

Osteomalacia is a disease of the bone metabolism that is characterized by defective mineralization of the bone matrix due to reduced phosphate levels. It presents with diffuse joint and bone pain, fractures, muscle wasting, limited mobility, weakness, and other nonspecific symptoms [1, 5, 8, 9].

In OO, fibroblast growth factor 23 (FGF23) is overexpressed by the tumor. It binds to the receptor on the proximal renal tubule and induces reduced expression of sodium phosphate co-carriers, leading to decreased renal phosphate reabsorption. It also inhibits the expression of 25-hydroxyvitamin D3 1-alpha hydroxylase, which results in inadequate production of 1,25(OH)_2_D, which is required for enteral absorption of calcium and phosphate [1, 5, 8, 9].

Laboratory results of those with hypophosphatemia show normal or decreased calcium, reduction of 1,25-dihydroxyvitamin D3 with resistance to vitamin D supplementation and high alkaline phosphatase [1, 8, 9].

Due to slow and indolent growth, as in this case, the symptoms of a metabolic disease became more evident than the nasal ones. Most tumors are detected when they are relatively small. This patient presented with a tumor that was 1.5 × 2.0 × 5.1 cm in size. This filled about two-thirds of the nasal cavity, which differs from those previously described [4, 10, 11].

The standard imaging examination for investigating OO and the tumor location is octreotide scintigraphy (Octreoscan). During this examination, the radiopharmaceutical binds to the somatostatin receptors that are expressed on the tumor surface [1, 8, 9].

A complementary imaging examination that can be used is tomography with contrast-enhancing and magnetic resonance. This can be useful for therapeutic programming and defining the surgical approach [5].

A definitive diagnosis was established by histopathological and immunohistochemistry [1]. Histologically, it is characterized by a prominent angiomatous pattern and the presence of perivascular fibroids, which are nonspecific. These may occur in hemangiopericytoma, glomus tumors, or angioleiomyoma [3].

Immunohistochemistry positive for SMA defines the diagnosis as it is possible to distinguish from other nasal tumors such as hemangiopericytoma, olfactory neuroblastoma, nasal glioma, and embryonic rhabdomyosarcoma [4].

The treatment is surgical and includes clinically normal tissue such as adjacent soft tissue. Most patients can be cured [1, 2, 10].

In the described cases of OO-inducing mesenchymal sinus tumors, 12 of the 13 cases had been completely cured. We reported the third case in which endonasal endoscopic surgery resulted in a favorable outcome [6].

Local recurrences have been described, although these have often been attributed to incomplete resection [1, 2, 10].

After exeresis, there is rapid clinical and laboratory recovery, with serum phosphorus returning to normal within the first 5 days in most patients. During this phase, the patient requires calcium supplementation to prevent hypocalcemia and hyperparathyroidism at a high level of 1,25(OH)_2_D [5].

## Conclusion

The presentation of a nasal tumor associated with osteomalacia is challenging diagnostically owing to the tumors that cause this condition. It is particularly rare for glomangioma to also present in the sinonasal region.

This case of glomangioma-induced osteomalacia suggests that surgeons and clinicians should consider sinonasal tumors as a differential diagnosis of osteomalacia.

Complete remission of OO and the absence of tumor recurrence with endoscopic resection gives greater validation to this technique. This may be useful for planning the treatment approach in future cases.

## Data Availability

Not applicable.

## Abbreviations

OO: Oncogenic osteomalacia
SMA: Smooth muscle actin
FGF23: Fibroblast growth factor 23
